# Impact of TRPV1 on Pathogenesis and Therapy of Neurodegenerative Diseases

**DOI:** 10.3390/molecules29010181

**Published:** 2023-12-28

**Authors:** Wenxin Wang, Tao Sun

**Affiliations:** Center for Precision Medicine, School of Medicine and School of Biomedical Sciences, Huaqiao University, Xiamen 361021, China; wwenxin999@126.com

**Keywords:** TRPV1, neurodegenerative diseases, agonists, antagonists, neuroinflammation

## Abstract

Transient receptor potential vanilloid 1 (TRPV1) is a transmembrane and non-selective cation channel protein, which can be activated by various physical and chemical stimuli. Recent studies have shown the strong pathogenetic associations of TRPV1 with neurodegenerative diseases (NDs), in particular Alzheimer’s disease (AD), Parkinson’s disease (PD) and multiple sclerosis (MS) via regulating neuroinflammation. Therapeutic effects of TRPV1 agonists and antagonists on the treatment of AD and PD in animal models also are emerging. We here summarize the current understanding of TRPV1’s effects and its agonists and antagonists as a therapeutic means in neurodegenerative diseases, and highlight future treatment strategies using natural TRPV1 agonists. Developing new targets and applying natural products are becoming a promising direction in the treatment of chronic disorders, especially neurodegenerative diseases.

## 1. Introduction

Neurodegenerative diseases (NDs) are complex disorders caused by damage to the neurons and glial cells in the central nervous system (CNS) or peripheral nervous system (PNS). The loss of neurons and the breakdown of neural networks lead to dysfunctions in cognitive behaviors, sensation and motor control, for instance, in Alzheimer’s disease (AD) and Parkinson’s disease (PD) [1,2]. Even though millions of people are affected by NDs, there are still no effective cures, mostly due to the lack of a thorough understanding of the pathogenesis of NDs.

In recent years, studies of a non-selective calcium channel, transient receptor potential vanilloid 1 (TRPV1), in the development of NDs have a significant attention [3,4,5,6,7]. TRPV1 is mainly expressed in the brain structures including the cerebral cortex, hippocampus, cerebellum, thalamus, central amygdala and the substantia nigra in rodents, as detected using various methods such as radioligand binding, immunohistochemistry, in situ hybridization, calcium imaging and slice electrophysiological recording [8,9] (Table 1). TRPV1 participates in regulating basic calcium signal transduction under physiological conditions, and acts as an “attack sensor” under adverse conditions [10,11]. The dysregulation of TRPV1 is associated with the occurrence and development of many neurological diseases, making TRPV1 a potential therapeutic target for treatment [10]. Accumulating evidence has suggested that TRPV1 is closely related to immune responses and might be recognized as a molecular switch in the neuroinflammation of the majority of neurodegenerative diseases [10].

In this review, we summarize preclinical evidence of the important role of TRPV1 channels in NDs with an emphasis on AD, PD and MS. We discuss the applications of using TRPV1 agonists and antagonists in the treatment of NDs. We highlight the reasoning of different discoveries of TRPV1 functions in various animal models of NDs and propose treatment strategies using natural TRPV1 agonists.

## 2. Structure and Function of TRPV1

**Figure 1:**
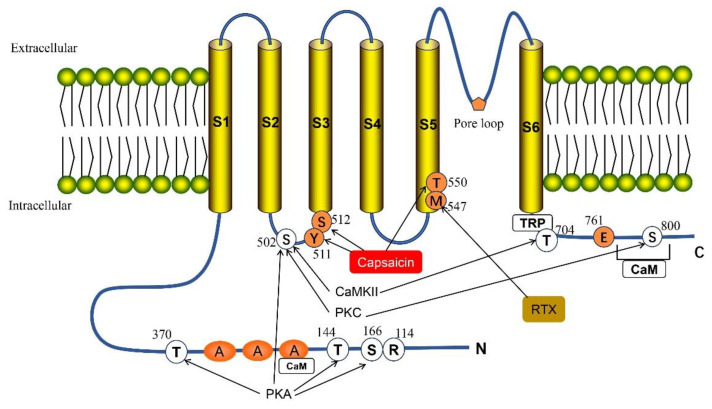
Structure of the full-length TRPV1. TPRV1 consists of three parts: the N- and C-terminus within the cell, six transmembrane regions (S1–S6) and the pore loop region formed between S5 and S6. TRPV1 has multiple phosphorylation sites: Thr 370, Thr 144, Ser 166 and Ser 502 can be directly phosphorylated by cAMP-dependent protein kinase (PKA). Protein kinase C (PKC) can phosphorylate two sites, Ser 502 and Ser 800. Ser 502 and Thr 704 are targets for calmodulin-dependent kinase II (CaMKII). Met 547 is responsible for the binding and sensitivity of resiniferatoxin (RXT), and also participates in some of the effects of vanillin. The C-terminal Glu761 and N-terminal Arg 114 are assumed to be agonist recognition sites. Ser 512 is important for capsaicin-mediated channel activation, while Thr 550 and Tyr 511 are necessary to maintain capsaicin sensitivity. There are two calmodulin-binding sites in the TRPV1 channel, one at the N-terminus and one at the C-terminus of the protein.

TRPV1 is the first identified member of the mammalian transient receptor potential superfamily with a wide expression in the brain, skin, visceral cells, muscles, intestines and pancreas [10,30,31]. The human TRPV1 protein is encoded by the *TRPV1* gene located in chromosome 17p13 [32]. TRPV1 forms a homo-tetramer channel structure, which resembles voltage-gated ion channels (VGICs) [33]. Each TRPV1 unit consists of six transmembrane regions (S1–S6) and a variable length of N and C terminals, with long tubular pore-shaped regions between S5 and S6 [32] (Figure 1). The central pore of the channel structure is surrounded by four independently folded S1–S4 regions, and the ion permeation pathway is conducted by transmembrane helices S5 and S6 [34,35]. The N terminal contains 3~4 connexin, which can connect different cytoplasmic proteins, and the C-terminal contains the phosphatidylinositol-4,5-diphosphate (PIP_2_) binding site and calcium ion/calcium regulatory protein binding site, which play an important role in signal transduction [36,37].

**Figure 2:**
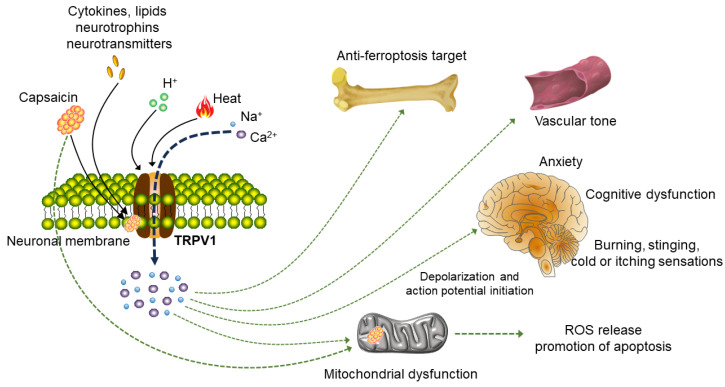
Signaling pathways acting on neurons and non-neuron cells after TRPV1 activation. TRPV1 can be activated by capsaicin, heat, protons, various cytokines, lipids, neurotrophins and neurotransmitters. Upon activation of plasma membrane TRPV1, different downstream signaling pathways will be induced in various cell types. The most studied cell type is neurons. After TRPV1 is activated, inflow of Na^+^ and Ca^2+^ can cause trigger action potential of nerve signal transmission and then stimulate various physiological activities of the body, participating in the generation and transmission of cold sensation, pain regulation and the regulation of inflammatory substances. Activation of TRPV1 also plays a role in the pathophysiology of neuropathic pain, anxiety and cognitive dysfunction. In addition, the TRPV1 channel is expressed in the human vascular system and affects vascular tension upon activation. In osteoarthritis, TRPV1 inhibits M1 macrophage polarization and reduces synovitis via Ca^2+^. Activating TRPV1 also can cause mitochondrial dysfunction and increase the release of ROS.

TRPV1 is a non-selective cation channel that can be activated by various physical and chemical stimuli, including heat (temperature > 43 °C), voltage, low PH (pH < 6) and vanillin [38,39]. It also can be activated by other lipid molecules, such as anandamide, N-arachidonoyldopamine and N-oleoyldopamine [40,41,42]. Once activated, the ion channel is opened, and permeability to calcium-based high-valence cations is increased, resulting in the flow of calcium ions into the cells to effectively depolarize them, generating action potential and stimulating various physiological activities [43,44] (Figure 2). Activation of TRPV1 can mediate the calcium influx, which increases the mitochondrial calcium and caspase activation and promotes the release of reactive oxygen species (ROS), leading to pro-apoptotic activity and neurotoxicity [10,45]. Reports have indicated that TRPV1 also is expressed in vascular smooth muscle cells [46]. Capsaicin activation of this channel demonstrates the potential for vasoconstriction, and suggests that the unnecessarily high consumption of capsaicin may lead to serious consequences, including vasospasm and myocardial infarction in potentially inflammatory patients [46]. Accumulated studies have shown that TRPV1 activation plays an important role in the generation and transmission of cold sensations, pain regulation, the pathophysiology of asthma, chronic pruritus, migraines, gastrointestinal motility disorders, anxiety and cognitive dysfunction [47,48,49,50,51] (Figure 2). Therefore, TRPV1 is considered a promising potential therapeutic target for the treatment of many human diseases [52].

## 3. TRPV1 and Neuroinflammation

In recent years, increasing evidence has shown that neuroinflammation is associated with neurodegenerative diseases such as Alzheimer’s disease and Parkinson’s disease. Microglia and astrocytes are key regulatory cells in the inflammatory response of the central nervous system [53]. It has been shown that TRPV1 is involved in microglia-induced inflammation [8,10].

**Figure 3:**
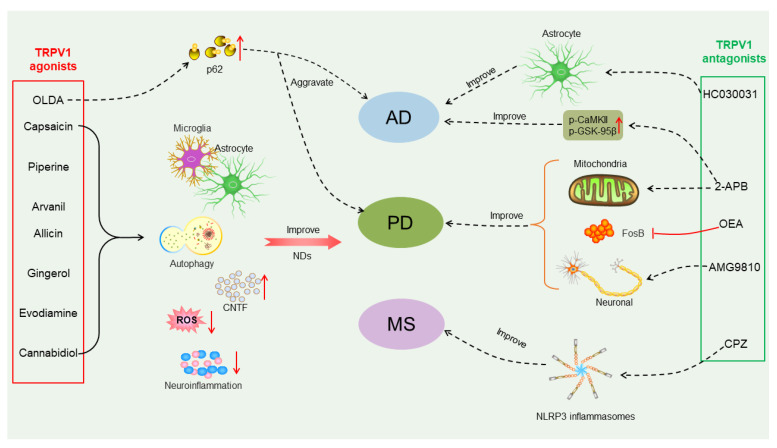
Different effects of TRPV1 agonists or antagonists on treatment of neurodegenerative diseases (NDs). TRPV1 agonists such as capsaicin, piperine, arvanil, allicin, gingerol, evodiamine and cannabidiol can improve NDs, including Alzheimer’s disease (AD), Parkinson’s disease (PD) and multiple sclerosis (MS), by acting on microglia or astrocytes, increasing autophagy, reducing neuroinflammation and reactive oxygen species production, mediating the production of ciliary neurotrophic factor (CNTF) and stimulating the body’s protective mechanisms. N-oleoyldopamin (OLDA) is also a TRPV1 agonist, and it enhances protein aggregation via regulating expression of p62/SQSTM1 and may exacerbate protein aggregation in AD and PD. TRPV1 antagonists 2-Aminoethoxydiphenyl borate (2-APB), oleoylethanolamide (OEA) and AMG9810 can be used to treat PD by rescuing mitochondrial dysfunction, inhibiting stromal FosB overexpression, and rescuing neuronal death. Among them, 2-ABP can also increase p-calmodulin dependent kinase II (CaMKII) and p-GSK-95β equal calcium to improve AD. TRPV1 antagonists HC030031 and capsazepine (CZP) can be used to treat AD and MS by acting on astrocytes and NLRP3 inflammasomes, respectively.

Studies have indicated that the stimulation of TRPV1 may exacerbate epilepsy, motor deficits in experimental autoimmune encephalomyelitis (EAE) and brain ischemia by activating the neuroinflammatory signaling pathway [54]. A recent study showed that TRPV1 is upregulated during neuroinflammation, and the TRPV1 antagonist capsazepine (CPZ) inhibits the activation of NLRP3 inflammasomes in the microglia, leading to reduced inflammatory infiltration and demyelination in EAE [55] (Figure 3). TRPV1 deficiency inhibited NLRP3 inflammasome activation in EAE mice and reduced central nervous system inflammation and microglial cell activation [55].

As expected, although some studies have shown that TRPV1 has pro-inflammatory effects, some experiments have indicated that TRPV1 plays a protective role in some neurological diseases. Reports have shown that TRPV1 exhibits protective effects in vascular dementia and Huntington’s disease by inhibiting oxidative stress and reducing the activation of ROS production in rat brains [10,56].

In summary, TRPV1 can regulate neuroinflammatory responses, suggesting potential therapeutic targets. These contradictory results may be caused by differences in TRPV1 agonists or antagonists, in particular their dosage and duration of use (Table 2).

## 4. TRPV1 and Alzheimer’s Disease (AD)

AD is the most common form of dementia and has a severe impact on one’s health and quality of life [57,58,59]. According to a report from the World Health Organization (WHO), approximately 50 million people worldwide suffer from dementia, and this number is expected to increase to 152 million by 2050 [60]. Amyloid plaques enriched with extracellular amyloid beta (Aβ) and intracellular neurofibrillary tangles comprising hyperphosphorylated Tau proteins are two major pathological features of AD [57,58]. To date, AD is still a serious fatal disorder for which effective treatments are meager.

Recent studies have shown that TRPV1 is closely associated with the pathogenesis of AD and is a potential therapeutic target for AD. Studies have indicated that changes in calcium signal transduction are one of the earliest events in AD [61]. Brain tissue from patients with AD has shown a significantly altered expression of Ca^2+^-handling genes, regulators of G protein signaling 4 and type B inositol 1,4,5-trisphosphate 3-kinase B [61]. It was found that knocking out the *TRPV1* gene can reduce the Aβ and Tau protein accumulation and rescue memory deficits in a 3×Tg-AD mouse model [62]. A study on the treatment of AD has shown that acute blockade of the TRPA1 channel with HC030031 (5 mg/kg body weight) restores the astrocyte activity to physiological levels, and can completely reverse early neuronal over-activity in a 1-month-old APP/PS1-21 mouse model of AD [63] (Figure 3). Daily intraperitoneal administration of the specific inhibitor of TRPA1 channel HC030031 from the 14th day of birth to 3 months of age in APP/PS1-21 mice inhibited the functional and structural changes in the astrocytes and neurons in the APP/PS1-21 mice. Moreover, chronic administration of the TRPA1 channel inhibitor HC030031 to the APP/PS1-21 mice promoted the compaction of amyloid fibrils into plaques, reduced plaques’ toxic effect and prevented spatial working memory defects in these mice, suggesting that chronic treatment with the TRPA1 inhibitor has a strong beneficial effect on Alzheimer’s disease progression at multiple levels [63]. Interestingly, the opposite effects of TRPV1 also have been observed in AD mouse models. Knockout of the *TRPV1* gene in a mouse model in which the murine *ApoE* gene locus was replaced by the human *APOE3* or *APOE4* gene exacerbated Tau pathology and recognition and memory impairments [64]. Thus, thorough studies, in particular on the dosage and duration of TRPA1 blockers, are required to draw more research efforts in the future.

Furthermore, research has shown that excessive neuronal activity may be a key feature of early AD [65]. Amyloid-β-dependent TRPA1 channel activation triggers hippocampal astrocyte hyperactivity, subsequently inducing hyperactivity in nearby neurons [66,67]. And this is crucial to promote cognitive decline and disease progression [68]. Interestingly, studies also have shown that capsaicin (1 mg/kg) rescues memory impairment, Tau pathology and neuronal autophagy dysfunction in ApoE4 high-fat-diet-fed mice. The activation of TRPV1 decreased neuronal lipid droplet accumulation and induced the upregulation of microglial phagocytosis of the synapses in ApoE4 mice [64]. Moreover, 7-month-old 3×Tg-AD mice treated with capsaicin (1 mg/kg) for one month showed decreased amyloid and phosphorylated Tau pathology, with reversed memory deficits due to the promotion of microglia activation, metabolism and autophagy [3]. In addition, studies have shown that capsaicin rescues the Aβ-induced degradation of hippocampal gamma oscillations by reversing both the desynchronization of action potential firing in CA3 pyramidal cells and the shift in the excitatory/inhibitory current balance [69,70,71]. Thus, it appears that activated TRPV1 plays a beneficial role in AD.

The following reasons might explain the different effects of TRPV1 in treating AD: First, the different AD mouse models used in the above studies have distinct pathological processes, which might respond differently to the activation or inhibition of TRPV1. Second, it has been shown that TRPV1 displays a bidirectional regulation of neuroinflammation [72]. It might cause a positive or negative effect on AD pathogenesis. Third, TRPV1 might be activated or suppressed in different subsets of cells in those AD mouse models [10]. Fourth, the dosage and duration of TRPV1 agonists vary among different AD animal models, which might lead to different therapeutic effects. Nevertheless, TRPV1 is becoming an innovative target for treating AD, with examinations of its biological functions and cell contact in the brain.

## 5. TRPV1 and Parkinson’s Disease (PD)

Parkinson’s disease is the second most common neurodegenerative disease, featuring the degeneration and death of dopaminergic neurons in the substantia nigra of the midbrain, and a reduction in the dopamine (DA) content in the striatum [73,74]. Driven by an increasing aging population and continuous environmental pollution, the global burden of PD is becoming demanding [75]. Ca^2+^ plays a central role in the normal functions of neurons and also is involved in many cellular processes such as oxidative stress, mitochondrial damage, proteasome dysfunction, excitatory toxicity, neuroinflammation and cell apoptosis [76]. An imbalance of calcium can lead to alterations in intracellular signaling cascades, and excessive calcium influx can greatly promote the development of PD [77]. Because TRPV1 is a non-selective cation channel with high permeability to calcium ions, particularly, it is expressed in brain regions, for instance, the striatum and substantia nigra, known to be affected in PD. Thus, TRPV1 is a highly relevant molecular target for developing new drugs for treating PD [78].

A study using the selective TRPV1 blocker AMG9810 (10 nmol) for the treatment of PD showed that AMG9810 could attenuate motor deficits in PD rat models after 6-hydroxydopamine (6-OHDA) administration and reduced neuronal death in the substantia nigra pars compacta of PD rat models [79] (Figure 3). Similarly, another TRPV1 blocker, capsazepine (5 mg/kg), also exhibited neuroprotective effects in a PD mouse model [80]. Moreover, studies have shown that the long-term use of L-DOPA in PD patients results in the development of abnormal involuntary movements called L-DOPA-induced dyskinesias [81]. Interestingly, the administration of oleoylethanolamide (5 mg/kg), an antagonist of TRPV1 receptors, reduced striatal FosB overexpression, and alleviated the intense axial, forelimb and orolingual dyskinetic symptoms and contralateral rotations caused by L-DOPA chronic treatment in the hemi-parkinsonian PD mouse model without interfering with the therapeutic effect of L-DOPA [82] (Figure 3).

Moreover, capsaicin is a highly selective agonist of the TRPV1 channel, and capsaicin (1 mg/kg) has been shown to restore dopamine signaling in a PD mouse model induced by 1-methyl-4-phenyl-1,2,3,6-tetrahydropyridine (MPTP), preventing MPTP-induced glial cell activation in PD mice and reducing oxidative stress in the astrocytes [83]. It is likely that the neuroprotective effects of TRPV1 are achieved via regulating the levels of the endogenous ciliary neurotrophic factor (CNTF) and CNTF-α receptors [84] (Figure 3). The direct use of capsaicin as a TRPV1 agonist in clinical practice is limited by its toxic side effects [85]. In particular, uncontrolled continuous stimulation of capsaicin can lead to the excessive influx of Ca^2+^ into the microglia, damage the mitochondria and even lead to cell apoptosis [4]. Therefore, controlling the influx of Ca^2+^ under stimulation is crucial. A recent study has shown that Cu2-x-Se-anti-TRPV1 nanoparticles can target the microglia and open a TRPV1 channel on its surface under second near-infrared (NIR-II) laser irradiation, and cause an influx of Ca^2+^ to activate the autophagy protein 5 (ATG5) and Ca^2+^/CaMKK2/AMPK/mTOR signaling pathways, which promote the phagocytosis and degradation of α-Syn, and improve treatment for PD [4].

In summary, TRPV1 agonists and blockers can effectively improve PD symptoms. Control of their dosage and usage time is required in future studies.

## 6. TRPV1 and Multiple Sclerosis (MS)

MS is a chronic neurodegenerative autoimmune disease of the central nervous system, which has a serious impact on the health of millions of people worldwide [86]. Experimental autoimmune encephalomyelitis (EAE) is one of the most widely used animal models to study MS. Studies have shown that the deletion of TRPV1 can alleviate EAE in mice and reduce neuroinflammation by inhibiting the activation of the NLRP3 inflammasome [55]. Deletion of the TRPV1 channels in *TRPV1* knockout mice exacerbated the defects of glutamate transmission occurring in the peak phase of EAE, and attenuated alterations in the GABA synapses in the chronic phase of EAE, in consistency with the dual effect of TRPV1 deletion on the motor deficits of EAE mice, suggesting a potential therapeutic target of TRPV1 for MS [54].

## 7. Agonists and Antagonists of TRPV1

Because TRPV1 has shown strong associations with the pathogenesis of neurodegenerative diseases, TRPV1 agonists and antagonists are becoming promising therapeutic targets (Figure 3, Table 2). The potential efficacy of certain TRPV1 agonists and antagonists also works via other receptors, including peroxisomes and peroxisome proliferator-activated receptor-α (PPAR-α), G protein coupled receptor (GPCR) and cannabinoid receptor [87,88]. In addition, these agonists or antagonists have different affinities to the receptors, so the off-target effects may also lead to the opposite outputs when using TRPV1 agonists and antagonists for treatment.

**Table 2:** Applications of TRPV1 agonists and antagonists in treatment of NDs.

Name	Binding Site	Function	Dosage	References
Endogenous agonists				
N-arachidonoyldopamine	CaM	May cause neurodegeneration	5 μM	[89,90]
N-oleoyldopamine	CB1R and CB2R	Activation of central histaminergic neurons	1 μM	[91]
Leukotriene B4	/	Increased amyloid-β formation	50 nM	[92]
Oleoylethanolamide	PKC	Improves AD- and PD-related pathology	5 mg/kg	[36,82,93]
Palmitoylethanolamide	PPAR-α	Offsetting neuroinflammatory conditions	100 mg/kg	[87]
Exogenous agonists				
Capsaicin	Y512, S513, T551 and E571	Alleviates pathological progression of NDs	1 mg/kg	[36,94]
Anandamide	Y511, S51 and R591	Preventing STZ-induced cognitive impairment	100 ng	[36,95]
Piperine	T671	Beneficial effects on NDs	5, 10 or 100 mg/kg	[96,97,98]
Gingerol	S4–S5 (T551 and E571)	Potential role in preventing NDs	10 mg/kg	[99]
Evodiamine	S510, T511, L515, T555, M568, I569, G570 and L571	Inhibition of neuroinflammation	100 mg/kg	[100,101]
Cannabidiol	Channel pore region	Beneficial protective role	10 mg/kg	[102]
Allicin	C157	Improves learning and memory	10 mg/kg	[103,104]
Arvanil	/	Alleviates hyperkinesia typical of HD	2 mg/kg	[105]
Endogenous antagonists				
Resolvin D2	GPR2	Restoration of nerve damage in PD rat model	25, 50, 100 ng/kg	[106]
Exogenous antagonists				
Caffeic acid	/	Improves AD- and PD-related pathology	5 and 10 mg/kg	[107,108]
I-RTX	/	Inhibiting ROS production	100 nM	[109]
SB-366791	L511, L515 L547, T550 and L553	Inhibits release of the pro-inflammatory sensory neuropeptide substance P	1 mg/kg	[110,111]
AMG 9810	M547 and L547	Attenuates hypokinetic effects of 6-OHDA	10 nmol	[79,112]
2-APB	K571	Improving mitochondrial dysfunction	10 mg/kg	[113,114]
Capsazepine	L515, V518, M547, I573 and L669	Improving inflammation in EAE mice	30 mg/kg	[80,114]
HC030031	/	Prevents neuronal dysfunction in AD mouse model	5 mg/kg	[63]

Capsaicin is a compound found in chili peppers, and is the most common natural TRPV1 agonist, which has high affinity, sensitivity and selectivity toward TRPV1. Although it may cause unpleasant sensations when consumed in large quantities, capsaicin has significant benefits in anti-inflammatory properties, obesity, cardiovascular and gastrointestinal diseases and various cancers when used at the correct dosage and frequency [115]. Therefore, the impact of spicy food consumption on health also has received widespread attention in recent years. Epidemiological studies have shown that the intake of spicy foods may have beneficial effects on hypertension, gastric ulcers, inflammatory bowel disease and gastrointestinal cancer [116,117,118]. And it is negatively correlated with the risk of death caused by cancer, ischemic heart disease and respiratory diseases [119]. On the contrary, it may be a risk factor for abdominal obesity [117].

**Figure 4:**
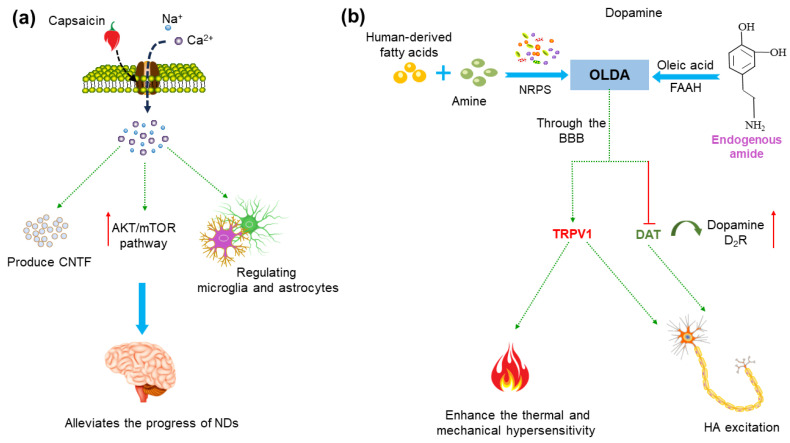
Potential mechanisms of treatment of neurodegenerative diseases (NDs) using capsaicin and N-oleoyldopamin (OLDA). (**a**) Capsaicin activates the TRPV1 receptor and Ca^2+^ influx, which can induce the upregulation of microglia phagocytosis, regulate its metabolic process and inhibit oxidative stress in microglia and astrocytes. It also can mediate the endogenous production of ciliary neurotrophic factor (CNTF), thereby stimulating activity of tyrosine hydroxylase (TH) enzyme and activating endogenous neuroprotective mechanisms. Activation of the TRPV1 channel upregulates the AKT/mTOR signaling pathway and reduces the production of inflammatory molecules. (**b**) OLDA in the human body comes from two pathways: one is the combination of dopamine and oleic acid under the action of fatty acid amide hydrolase (FAAH). Moreover, clostridium in the intestine can also bind foreign substrates (fatty acids) commonly found in the human body or diet with amines under the action of non-ribosomal peptide synthases (NRPS) to produce OLDA. OLDA can reach the brain via the blood–brain barrier (BBB), enhancing thermal and mechanical hypersensitivity by activating the TRPV1 receptor. OLDA activates TRPV1 and blocking dopamine transporter (DAT) and dopamine 2 receptor (D2R) activation, and then leads to a wake-on-firing pattern of histaminergic (HA) neurons, which may have beneficial effects on the treatment of PD.

Natural capsaicin has low toxicity and multiple biological functions, for instance, its good analgesic and anti-inflammatory effects, and its activation of the Ca^2+^-permeable TRPV1 channel, which can effectively cross the blood–brain barrier (BBB) [120]. The TRPV1 channel being activated by capsaicin can trigger an increase in the intramitochondrial Ca^2+^ concentration and mitochondria depolarization, resulting in the enhancement of chemotactic activity and autophagy in the microglia [3,64] (Figure 4a). This has played a beneficial role in the research of AD and PD.

Capsaicin can activate the AKT/mTOR pathway and reduce the production of inflammatory factors [121]. It also can mediate the production of ciliary neurotrophic factor (CNTF), concurrently stimulate tyrosine hydroxylase (TH) enzyme activity via the phosphorylation of TH, and contribute to functional recovery [84,122] (Figure 4a). Moreover, capsaicin can reduce Aβ and pTau pathology and improve the neurodegeneration and cognitive impairment of AD model mice by activating the TRPV1 channel [71,121,123,124,125]. Thus, accumulating evidence supports that capsaicin plays a beneficial role in AD and PD, and capsaicin-activated TRPV1 can reduce neuroinflammation, protect dopamine neurons and ultimately alleviate the progress of AD and PD [13,83,122,126,127,128,129] (Figure 4a).

Furthermore, N-oleoyldopamin (OLDA) is an endogenous lipid derivative condensed from oleic acid and dopamine [91] (Figure 4b). Studies have shown that the TRPV1 channel can be activated by OLDA, and in turn enhances the thermal and mechanical hypersensitivity induced by the proinflammatory mediator bradykinin (BK), suggesting that OLDA is an endogenous agonist of TRPV1 [40] (Figure 4b). OLDA modulated the firing of nigrostriatal DA neurons by interacting with TRPV1 and blocking the dopamine uptake (dopamine transporter, DAT) and dopamine 2 receptor (D2R) activation, then leading to a wake-on-firing pattern of histaminergic (HA) neurons, suggesting the beneficial role of OLDA in PD [91,130] (Figure 4b).

Interestingly, it also has been shown that OLDA functions as an aggresome formation inducer and leads to aggresome formation and protein aggregation without proteasome inhibition via regulating p62/SQSTM1 expression [131]. SQSTM1/p62 is a scaffold protein closely involved in the macroautophagy process. During the process of functional autophagy, p62 is usually degraded along with the substrate, so the accumulation or aggregation of p62 indicates impaired autophagy. Research has shown that p62 is accumulated in pyramidal neurons in the AD brain [132,133]. An increase in p62 and impaired autophagy were also observed in *APP* knockout AD model mice [133]. Moreover, it was found that autophagy failure leads to p62-related α-synaptic nuclear protein accumulation in PD studies [131]. This indicates that the abnormal clustering of p62 may represent the deterioration of neurodegenerative diseases. Therefore, OLDA, by regulating the p62 and autophagy function, has great potential as a promising target for the treatment of AD and PD.

**Figure 5:**
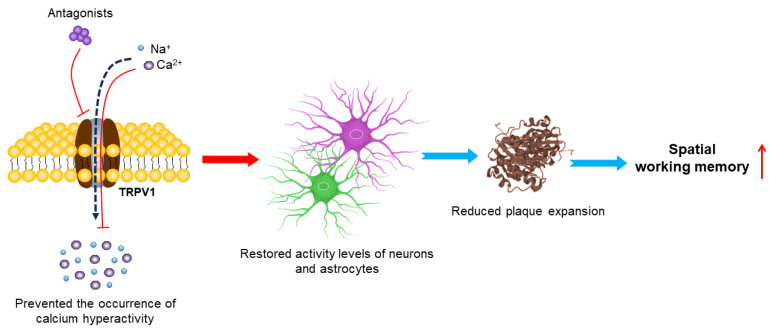
Mechanisms of TRPV1 antagonists in treating AD. Inhibition of the TRPV1 channel by its antagonist prevents occurrence of astrocyte calcium hyperactivity, restores neuronal and astrocyte activity levels and increases plaque compaction and diffusion. This early multi-level neuroprotection seems to prevent subsequent decline in spatial working memory.

In addition, the functions of TRPV1 antagonists also have been examined (Table 2). Studies have shown that the astrocytic calcium activity is dramatically increased, which is synchronous with nearby Aβ plaques in APP/PS1 double-transgenic mouse models of AD [134]. Acute blockade of the TRPV1 channel with the TRPV1 antagonist HC030031 displayed a full restoration of the astrocyte activity at the physiological levels and the complete reversal of early neuronal hyperactivity [62]. HC030031 pharmacological treatment also prevented the occurrence of hippocampal astrocytic calcium hyperactivity in APP/PS1-21 mice, and restored the neuron and astrocyte activity levels, protected the structural and functional neuronal integrity and reduced plaque expansion in the hippocampus and declines in spatial working memory [63] (Figure 5).

Moreover, the TRPV1 antagonist caffeic acid was shown to attenuate the loss of dopaminergic neurons, and to improve behavioral impairments in PD animal models [107,108]. The TRPV1 antagonist AMG9810 was observed to reduce neuronal death in the substantia nigra pars compacta, and to improve motor dysfunction in 6-OHDA-induced PD rat models [79] (Figure 3). 2-aminoethoxydiphenyl borate (2-APB) is also a well-known antagonist molecule with concentration-dependent effects on TRPV1. Recent studies have found that 2-ABP has neuroprotective effects on neurodegenerative diseases, including AD and PD (Figure 3) [113,135,136].

Therefore, consistent with the contradictory roles of the TRPV1 channel in the pathogenesis of AD and PD, its agonists and antagonists have been shown to play both positive and negative effects in treating AD and PD (Table 2). Comprehensive studies on the dosage effects of TRPV1 agonists and antagonists and more accurate animal models for NDs are required for future work.

## 8. Conclusions

Neurodegenerative diseases such as AD and PD are severe chronic disorders that are ultimately lethal. Accumulating studies have demonstrated significant associations of the TRPV1 channel with the pathogenesis of AD and PD. It is likely that the activation and inhibition of TRPV1 by their agonists and antagonists, respectively, modulate the activation, autophagy, phagocytosis and metabolic functions of the microglia and astrocytes, in turn modifying their oxidative stress and normal functions, and achieve the treatment of AD and PD by regulating neuroinflammation. In particular, natural TRPV1 agonists such as capsaicin and resin toxin (RTX) have shown promising effects in delaying neuronal cell death [137]. Moreover, piperine from black pepper, eugenol from cloves, curcumin from horseradish and gingerol from ginger also have been shown to activate TRPV1 receptors [138,139]. Thus, exploring the role of natural TRPV1 agonists will expand our means of treating chronic diseases and developing new medicines to improve human health.

## Figures and Tables

**Table 1:** Expression and function of TRPV1 in the CNS.

Species	CNS Area	Function	References
Rat	Substantia nigra	Mitochondrial damage and microglial cell damage	[12]
Mouse	Hippocampal	Involved in production of ROS in microglia	[13,14,15]
Mouse	Amygdala	May participate in controlling amygdala learning mechanism	[16,17]
Rat	Basal ganglia	Regulates dopamine transmission	[18]
Mouse	Nucleus accumbens	Mediates synaptic modification	[19]
Mouse	Striatum	May be related to dopamine pain	[20]
Mouse	Cortex	Participates in or counteracts pain	[21]
Mouse	Hypothalamus	Participates in controlling energy homeostasis	[22]
Mouse	Olfactory system	Involved in stimulating proliferation of progenitor cells	[23]
Mouse	Midbrain	Related to regulating psychomotor behavior	[24]
Rat	Brainstem	Associated with meningitis, abnormal pain and migraine	[25]
Mouse	Spinal cord	Related to nociceptive, inflammatory and neuropathic pain	[26,27]
Mouse	Retina	Increases translocation of IL-6 and NF-κB	[28]
Mouse	Trigeminal ganglia	Participates in mechanical hyperalgesia	[29]
